# Uric acid: a potent molecular contributor to pluripotent stem cell cardiac differentiation via mesoderm specification

**DOI:** 10.1038/s41418-018-0157-9

**Published:** 2018-07-23

**Authors:** Bingbing Ke, Yujie Zeng, Zhihong Zhao, Fusheng Han, Taoyan liu, Jingyi Wang, Anila Khalique, Wen-Jing Lu, James Chong, Feng Lan, Hua He

**Affiliations:** 1 Department of Emergency Cardiology, Beijing Anzhen Hospital, Capital Medical University, Beijing, 100029 China; 2 Department of Cardiology, ZhouPu Hospital, PuDong New Area District, Shanghai, 200120 China; 3 Beijing Lab for Cardiovascular Precision Medicine, Anzhen Hospital, Capital Medical University, Beijing, 10029 China; 4 Department of Ultrasound, Beijing Anzhen Hospital, Capital Medical University, Beijing, 100029 China; 5 Centre for Heart Research, Westmead Institute for Medical Research, The University of Sydney, Hawkesbury Rd, Westmead, NSW 2145 Australia; 6 Department of Cardiology, Westmead Hospital, Hawkesbury Rd, Westmead, NSW 2145 Australia

**Keywords:** Biocatalysis, Ubiquitylation

## Abstract

Congenital heart disease (CHD) is the most common cause of congenital anomaly and a leading cause of morbidity and mortality worldwide. Generation of cardiomyoctyes derived from pluripotent stem cells (PSCs) has opened new avenues for investigation of human cardiac development. Here we report that uric acid (UA), a physiologically abundant compound during embryonic development, can consistently and robustly enhance cardiac differentiation of human PSCs including hESCs and hiPSCs, in replacement of ascorbic acid (AA). We optimized treatment conditions and demonstrate that differentiation day 0–2, a period for specification of mesoderm cells, was a critical time for UA effects. This was further confirmed by UA-induced upregulation of mesodermal markers. Furthermore, we show that the developing mesoderm may be by directly promoted by SNAI pathway-mediated epithelial–mesenchymal transition (EMT) at 0–24 h and a lengthened G0/G1 phase by increasing the ubiquitination degradation in 24–48 h. These findings demonstrate that UA plays a critical role in mesoderm differentiation, and its level might be a useful indicator for CHD in early fetal ultrasound screening.

## Introduction

Congenital heart disease (CHD) is defined as a gross structural abnormality of the heart or great vessels and it is a major cause of morbidity/mortality in infancy and childhood [1–3]. In embryonic development, especially 3–8 weeks, cardiac development is vulnerable to various physical and chemical factors, which can lead to heart developmental abnormalities [4, 5]. At present, the basic fetal cardiac examination by ultrasound is usually performed between 18 and 22 weeks, during the second trimester of pregnancy [6]. Thus, diagnosis and treatment of congenital heart disease in the early pregnancy were very limited. Interestingly, our clinical data suggest that maternal abnormalies of uric acid (UA) might be a risk factor of CHD as shown in table S1.

Human induced pluripotent stem cells (hiPSCs) are increasingly used in various areas of cardiovascular research, including disease modeling, cardiotoxicity screening, drug discovery, and the study of human cardiac development [7]. The in vitro differentiation of hESCs into cardiomyocytes provides a unique model to study heart development. Ascorbic acid (AA) has been reported to enhance cardiac differentiation of murine iPSCs, potentially by increasing collagen synthesis that results in greater proliferation of cardiovascular progenitor cells [8]. Interestingly, removal of AA from chemically defined medium does not result in cell death, but merely in a reduction in cardiomyocyte yield [7].

To test the effects of UA on cardiac development, we systematically used UA in a dose-dependent manner to facilitate the cardiac differentiation of hESCs in vitro. We found that UA robustly and reproducibly enhanced cardiac differentiation of hESCs during early stage of differentiation. Then, we investigated the mechanisms underpinning UA-promoted cardiac differentiation and showed that UA could specifically promote mesoderm differentiation. Furthermore, we show this is caused by enhanced epithelialmesenchymal transition (EMT) within 24 h and by ubiquitination to lengthen the G1 phase of the cell cycle. Our results suggest that UA measurement of pregnant woman might be an early indicator for the detection of the CHD prior to ultrasound screening.

## Results

### UA consistently and robustly promotes cardiac differentiation of hESCs

**Fig. 1:**
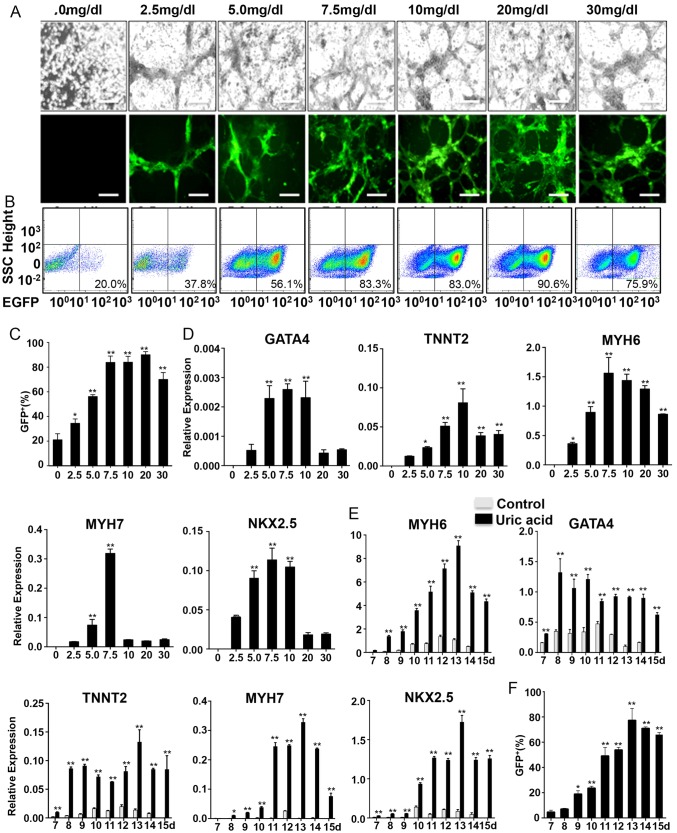
UA enhances cardiogenesis of hESCs. **a** NKX2–5-GFP hESCs were treated with the indicated concentration of UA for 15 days under inverted microscope. EGFP expression was analyzed under fluorescent microscope with a fixed exposure time. Scale bars = 200 μm. **b** Percentages of EGFP^+^ cardiomyocytes at day 15 in the total population derived from NKX2–5-GFP hESCs with or without UA treatment. **c** Percentages of NKX2–5-GFP hESCs-derived cardiomyocytes at day 15 with different concentration of UA. The results were plotted as percentages of the unstimulated levels. **d** Percentages of NKX2–5-GFP hESCs-derived cardiomyocytes from d7 to d15. **e** NKX2–5-GFP hESCs were treated with 7.5 mg/dl UA for 15 day. The expression of GATA4, TNNT2, NKX2–5, MYH6, MYH7, and GADPH was examined with quantitative PCR, and the results were expressed as relative expression to GADPH and plotted as percentages of the maximum. **f** Time windows for UA-promoted cardiac differentiation in cells treated with or without 7.5 mg/dl UA. The expression of GATA4, TNNT2, NKX2–5, MYH6, MYH7, and GADPH was examined with kinetic PCR, and the results were expressed as relative expression to GADPH and plotted as percentages of the maximum. *n* = 3 each. Data are expressed as means ± SD. *P < 0.05, **P < 0.01 vs. control

To characterize the effect of UA on the cardiogenic differentiation of PSCs, NKX2–5-GFP ESCs were treated with UA from 2.5 to 30 mg/dl for 15 days from the initiation of differentiation. The percentage of EGFP^+^ cells by flow cytometry and the relative expression level of cardiac gene *GATA4*, *TNNT2*, *NKX2–5*, *MYH6*, *MYH7* significantly increased in an inverted U-shaped manner and reached a peak around 7.5–10 mg/dl (Fig. 1a, b, c, e). There were no viable cells in plates without UA. We performed flow cytometry with these dead cells to confirm that the ~ 20% GFP positive background was from autofluorescence. Owing to precipitation of UA at high concentrations, we choose 7.5 mg/dl as an optimum concentration in the subsequent experiment. Then we further examined the profiles of cardiac induced cells with or without UA. Spontaneously beating cardiomyocytes emerged around day 9 (Supplemental Movie 1) with UA treatment. Expression of the cardiac genes *GATA4*, *TNNT2*, *NKX2–5*, *MYH6*, *MYH7* increased significantly as compare to without UA treatment. (Fig. 1d, f), implying consistent and robust UA-promoted cardiogenesis.

### UA promotes cardiac differentiation in multiple stem cell lines

**Fig. 2:**
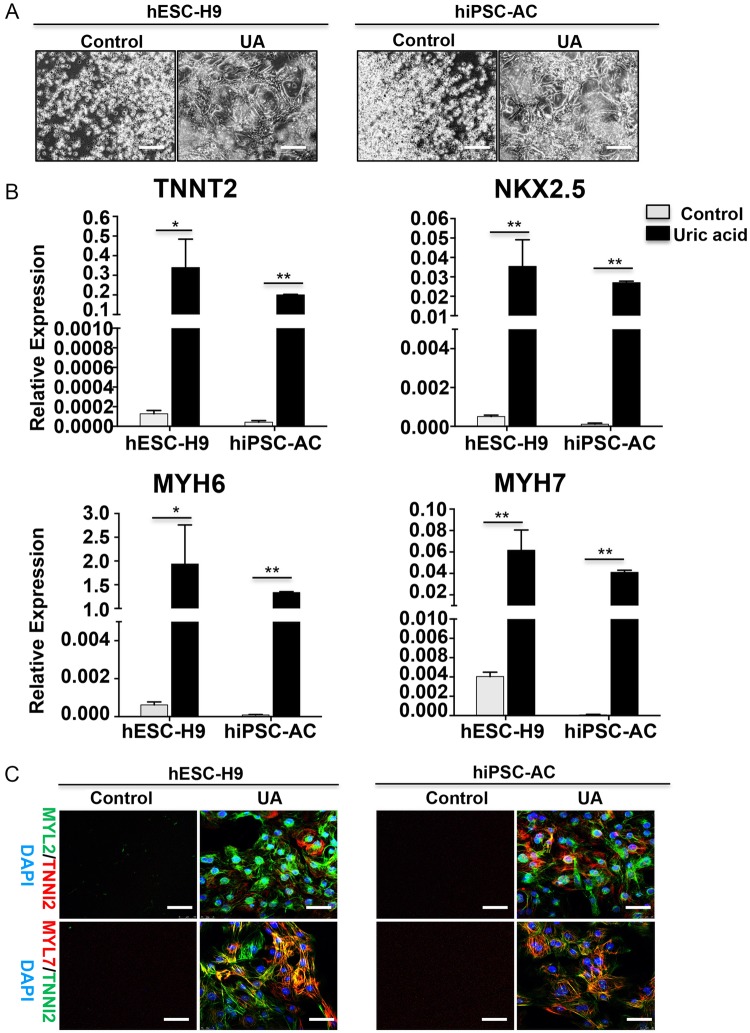
UA induced cardiomyocyte differentiation in other two PSCs lines. **a** Two PSCs lines were induced in the development of cardiomyocytes with or without 7.5 mg/dl UA. Data were collected at day 15. **b** Quantitative RT-PCR analysis of the expression level of cardiac genes. **c** Immunostaining showing emergence of MYL2^+^, MYL7^+^, and TNNI2^+^ cardiomyocytes in UA-treated or not cardiomyocytes at day 15. Scale bars = 200 μm. Nuclei were counterstained with DAPI (blue). *n* = 3 each. Data are expressed as means ± SD. *P < 0.05, **P < 0.01 vs. control

Stem cell lines may display a huge variation in their cardiac differentiation potential. To confirm the pro-cardiogenic effect of UA in different stem cell lines, we then tested UA on hESC-H9 and SFhiPSC lines using the same methods described above and profiled the resulting cardiac populations. Consistently, robust beating was observed in UA groups (Fig. 2a). The cardiac genes NKX2–5, TNNT2, MYH6, MYH7 were also robustly increased in UA-treated cells during differentiation (Fig. 2b) and the presence of MYL2^+^, MYL7^+^, or TNNI2^+^ cardiomyocytes were only detected in UA-treated cells at day 15 from all stem cell lines (Fig. 2c). These data confirm that UA can support cardiac differentiation in hESC and hiPSC lines in vitro.

### UA promotes cardiac differentiation in the early stages of development

**Fig. 3:**
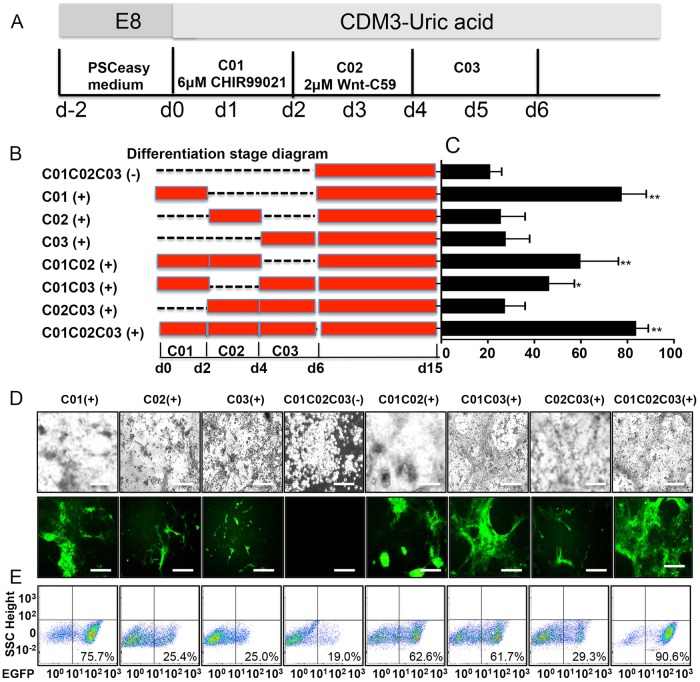
UA promoted cardiac development in the early stage of differentiation. **a** Schematic of cardiac differentiation. C01: CDM3-UA + CHIR99021; C02: CDM3-UA + Wnt-C59; C03: CDM3-UA. D, day. **b** Schematic diagram of the differentiation protocols. **c** Efficiency of cardiac differentiation measured by flow cytometry for EGFP^+^ on day 15 cells in different differentiation protocols. The entire group compared with the group of C01C02C03 (-). **d** NKX2–5-GFP hESC cells were treated with differentiation protocols for 15 days under inverted microscope. EGFP expression was analyzed under fluorescent microscopy with a fixed exposure time. Scale bars = 200 μm. **e** Percentages of EGFP^+^ cardiomyocytes at day 15 in the differentiation protocols. *n* = 3 each. Data are expressed as means ± SD. *P < 0.05, **P < 0.01 vs. control

To determine the specific function of UA at exact time points, we induced UA at different stages of cardiogenesis (Fig. 3a). In brief, we added 7.5 mg/dl UA during early (day 0–2), mid (day 2–4), and late (day 4–15) phases of hESC differentiation or various combinations (Fig. 3b). UA treatment during differentiation day 0–2 only resulted in cardiac differentiation efficiency of 77.6% ± 8.57 by flow cytometry, which is comparable to UA treatment throughout the entire differentiation period. In contrast, withdrawal of UA during day 0–2 almost prevented cardiogenesis (Fig. 3c–e). Moreover, although withdrawal of UA during day 2–4 or 4–6 significantly reduced the efficiency of cardiac differentiation, both conditions were capable of generating cardiomyocytes (Fig. 3c–e). AA was previously reported to robustly enhance cardiogenesis from hESCs by promoting the proliferation of cardiac progenitor cells at differentiation day 2–6 in a collagen synthesis-dependent mechanism [8]. With chemically defined conditions, we confirmed that AA could robustly promote cardiomyocyte generation except for withdrawal at differentiation day 2–4 (Fig. 3a and S1), which was consistent with previous study. *P* value between C01C02C03 (-) and others shown in Fig. 3c, and the *P* value of all comparisons was shown in table S2. These results reveal that the early-phase (day 0–2), a critical phase for mesoderm development, is the most crucial period for UA to exert the pro-cardiogenic effects. We then examined the profiles of NKX2–5-GFP^+^ cells with or without UA treatment. Upon induction, the majority of NKX2–5-GFP hESCs without UA treatment were dead and by day 15 no GFP^+^ cells were visible (Fig. 3d). However, flow cytometry analysis showed ~ 20% expression of GFP in the remaining small fraction of cells in day 0–2 UA withdrawal groups (Fig. 3e). Therefore, we considered ~ 20% to be the baseline of cardiac differentiation causing autofluorescence of dead cells/debris. We found either UA or AA could promote cardiomyocyte differentiation, respectively. We then further examined whether UA enhanced cardiomyocyte differentiation efficiency in cardiogenic conditions. UA and AA were added into medium simultaneously and induced for 15 days. CDM3 without AA was used as the control group for non-cardiogenic differentiation. We found no difference in cardiomyocyte differentiation efficiency with UA or AA (Figure S1 C). As UA promoted cardiac differentiation at 0–48 h but not AA, we further examine the effects of AA on mesoderm genes as well as EMT and G1 phase-related genes. As shown in the Figure S1 D, E, cells treated with or without AA expressed similar level of GSC, MIXL1, CDH1, VIM and CCDN1 at both 24 and 48 h. Thus, AA does not promote EMT and lengthened G1 phase. To further compare the pro-cardiogenic mechanism of UA and AA, we examined their impact on the 2–4 days, which is critical for the development of cardiac mesoderm. As AA was reported to promote the proliferation of cardiac progenitor cells (CPCs) at 2–6 days of differentiation, and promote collagen synthesis through the MEK-ERK1/2 pathway [8]. We examined the CPC genes, NXK2–5 and TBX-5, when UA or UA was added at day 0–2 or day 2–4, respectively (Figure S3 E). We found that most cells were dying in C02-UA (+), C01-AA (+), and control groups, whereas C02-UA (+) cells proliferated robustly (Figure S3 F). Moreover, only C02-AA (+) and C01-UA (+) cells showed increased expression of NXK2–5 and TBX-5 (Figure S3 G).

### Effect of antioxidants vs. UA in cardiac differentiation

**Fig. 4:**
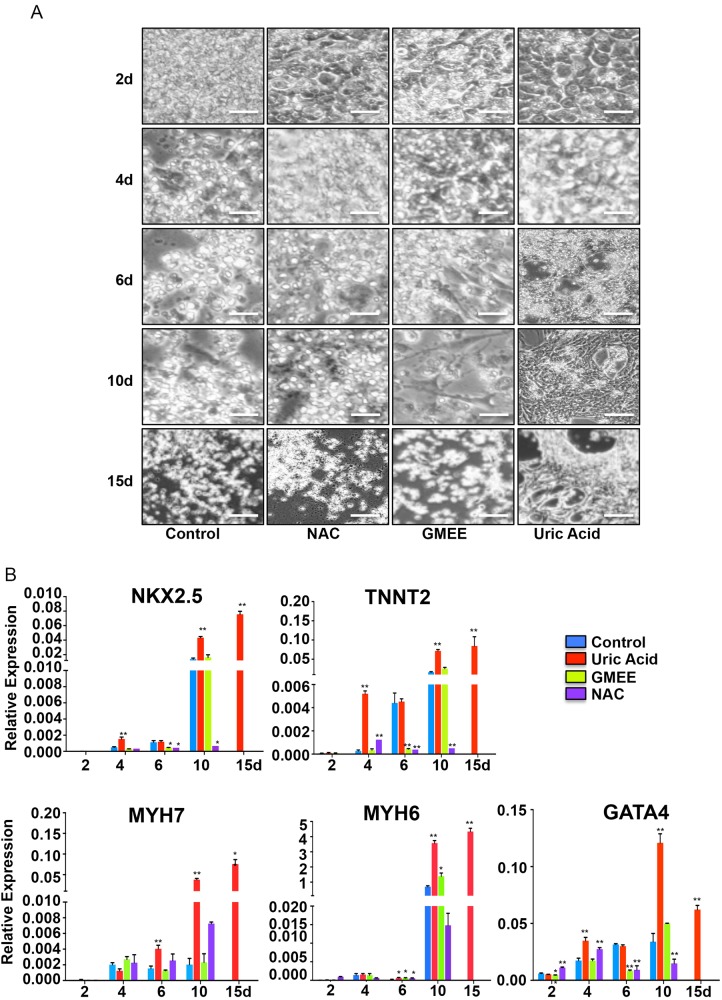
Antioxidants failed to mimic the effect of UA-induced cardiac differentiation. **a** NKX2–5-GFP hESCs were treated with or without 7.5 mg/dl UA, 10*3 mol/L NAC, or 10*4 mol/LGMEE. **b** The expression of GATA4, TNNT2, NKX2–5, MYH6, and MYH7 were examined with quantitative PCR, and the results were expressed as relative expression to GADPH and plotted as percentages of the maximum. *n* = 3 each. Data are expressed as means ± SD. *P < 0.05, **P < 0.01 vs. control

As biological effects of UA are often attributed to its antioxidative properties, we replaced UA with typical antioxidants such as N-acetyl-L-cysteine (NAC) and reduced gluthatione (GMEE). These failed to recapitulate the effects of cardiac differentiation observed with UA based on morphology (Fig. 4a) or the expression of major cardiac specific genes (GATA4, TNNT2, NKX2–5, MYH6, and MYH7) (Fig. 4b). These results suggest that the cardiogenic differentiation promoted by UA is independent of its antioxidative properties.

### Analysis of transcriptional profiles

**Fig. 5:**
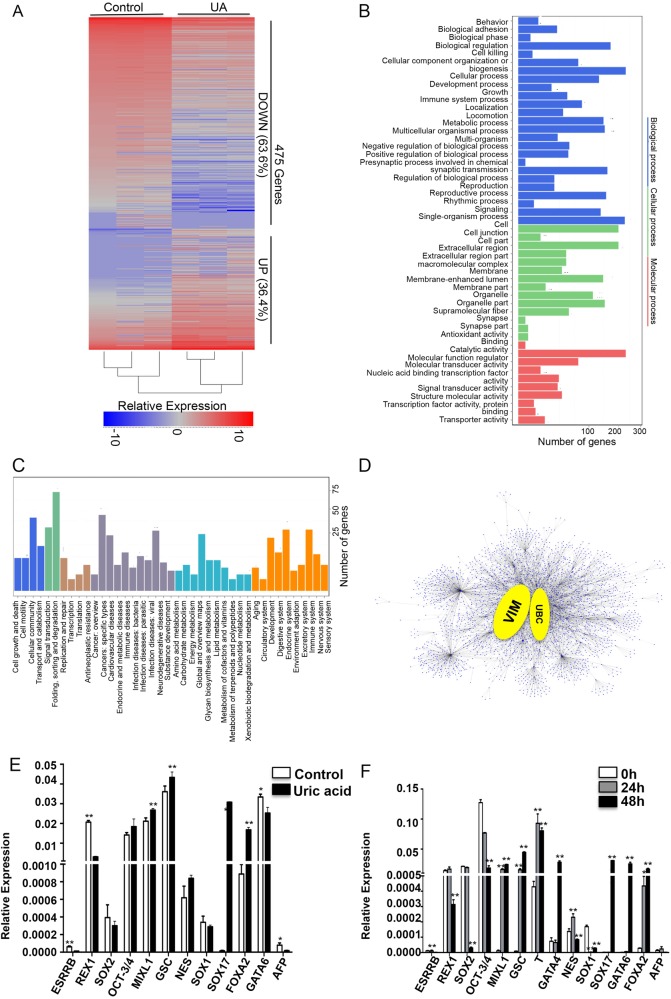
Analysis of transcriptional characteristics with or without UA treatment. **a** Heatmap shows hierarchical clustering of differentially expressed genes with or without UA treatment. Values are row-scaled to show relative expression. Blue and red are low and high levels, respectively. Representative down- (blue box) and upregulated (red box) genes are listed. **b** Gene ontology (GO) term categorization and distribution of differentially expressed genes. GO terms were processed and categorized under three main categories (cellular component, molecular function, and biological process). **c** KEGG pathway Rich detail figure. **d** Protein–protein Interaction Network of DEGs. **e** Quantitative PCR validation of certain DEGs, including the genes of pluripotent, endoderm, mesoderm, and ectoderm. **f** The expression of pluripotent, endoderm, mesoderm, and ectoderm were examined with quantitative PCR at UA treatment for 0, 24, and 48 h. *n* = 3 each. Data are expressed as means ± SD. *P < 0.05, **P < 0.01 vs. control

To explore the functional consequences of gene expression changes caused by UA exposure, we performed RNA-seq analysis on cells at differentiation day 2 with or without UA. We found 475 genes that were significantly expressed out of which 173 genes upregulated and 302 downregulated after UA treatment. (Fig. 5a).

To understand the biological mechanisms underpinning the effects of UA, we performed GO enrichment analysis of 475 differentially expressed genes (DEGs). In total, 14, 10, and 25 categories were identified in cellular component, molecular function, and biological process groups, respectively (Fig. 5b). Moreover, differential expression in the binding group of molecular function were increased up to 52.4%, whereas differential expression in the cell process, single-organism process, biological regulation groups of biological process were 11.6%, 11.4%, and 8.5%, respectively. GO enrichment analysis of DEGs showed that single-multicellular organism process, multicellular organism development, anatomical structure development, pattern specification process, anatomical structure morphogenesis, developmental process, embryo development, and single-organism developmental process were the most remarkable groups within biological process (Fig. 5b), which indicated UA can promote developmental processes in early stages of differentiation.

To understand the biological impacts of DEGs, we performed Kyoto Encyclopedia of Genes and Genomes (KEGG) analysis. A total of 209 pathways with significantly differential expression were identified between the treatment group and control, of which 8 pathways showed significant differences (*P* < 0.05, Fig. 5c). These pathways were mainly related to cellular communication, development, and signal transduction.

To identify DEGs playing key regulatory roles, we applied cytoscape version 3.4.0 to analyze the regulatory network based on a large set of gene expression data. We found that VIM and UBC genes might be the master players during early development (Fig. 5d).

To elucidate the critical stage for UA to promote cardiac differentiation of hESCs, we selected genes of pluripotency as well as endoderm, mesoderm and ectoderm markers from RNA-seq in figure S2. The pluripotent genes, ESRRB, REX1, were downregulated, whereas endoderm (SOX17 and FOXA2) and mesoderm (MIXL1 and GSC) genes were upregulated compared with controls. There was no change in ectoderm genes (NES and SOX1). We next confirmed this result by real-time reverse transcriptase-polymerase chain reaction (RT-PCR) (Fig. 5e). After accessing these genes at 0, 24, and 48 h we found pluripotency (ESRRB and REX1) and ectoderm genes (NES and SOX1) gradually downregulated, whereas mesoderm (MIXL1, GSC, and GATA4) and endoderm (SOX17, FOXA2, and GATA6) genes were gradually upregulated after UA treatment. These results reveal that UA can support mesoderm differentiation of embryonic stem cells. Taken together with results of UA during differentiation day 0–2 (Fig. 3c), these data suggested that UA increases mesoderm development.

### UA promotes cardiac differentiation through increased EMT within 0–24 h

**Fig. 6:**
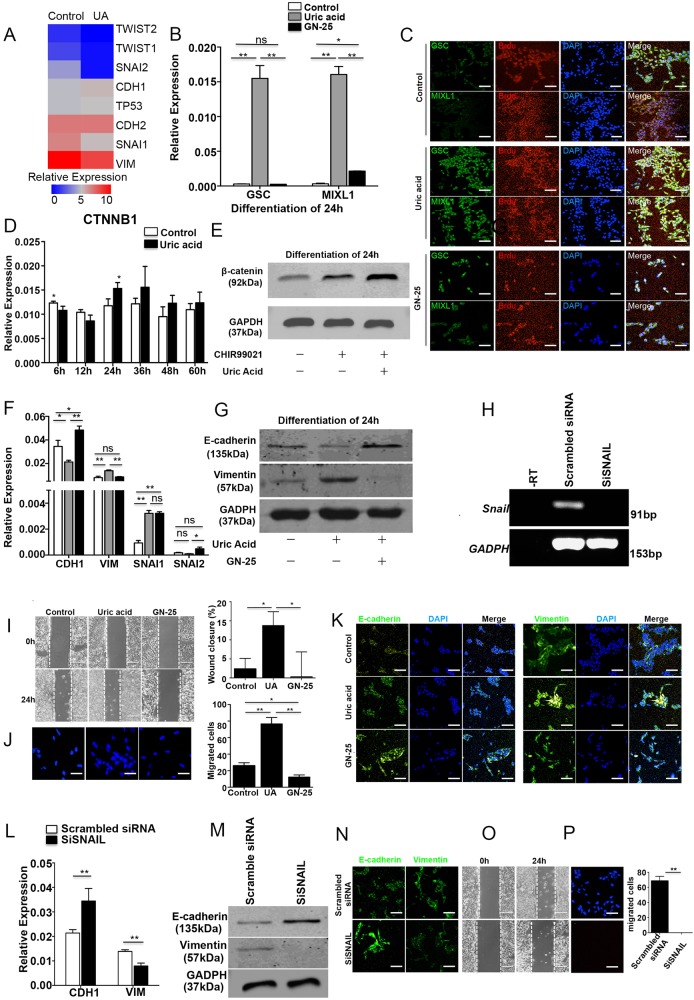
UA promoted cardiac differentiation through increasing EMT. **a** Heatmap shows hierarchical clustering of EMT genes. **b** The expression of mesoderm genes, GSC and MIXL1, were examined with quantitative PCR with or without UA and GN-25. The results were expressed as relative expression to GADPH and plotted as percentages of the maximum. **c** Immunostaining of GSC or MIXL1 and BrdU in 24 h. Scale bars = 200 μm. **d** The expression of CTNNB1 was examined with quantitative PCR with or without UA for every 12 h. The results were expressed as relative expression to GADPH and plotted as percentages of the maximum. **e** Immunoblot analysis of beta-catenin in 24 h. The results were expressed as relative expression to GADPH. **f** The expression of EMT genes, VIM, CDH1 SNAI1, and SNAIL2 were examined with quantitative PCR with or without UA and GN-25. The results were expressed as relative expression to GADPH and plotted as percentages of the maximum. **g** Immunoblot analysis of e-cadherin and vimentin in 24 h. The results were expressed as relative expression to GADPH. **h** Gel electrophoresis with SNAIL1 SiRNA. The results were expressed as relative expression to GADPH. (**i**) NKX2–5-GFP hESCs with or without UA treatment was analyzed in an in vitro wound model. Scale bars = 200 μm. The percent wound closure (%) was counted under a light microscope. **j** Transwell assay in vitro. Migration cell was counted by DAPI in fluorescence microscope. Scale bars = 200 μm. **k** Immunostaining of e-cadherin and vimentin with or without UA and GN-25 for 24 h. Scale bars = 200 μm. **l** The expression of EMT genes, VIM and CDH1, were examined with quantitative PCR with SiRNA. The results were expressed as relative expression to GADPH and plotted as percentages of the maximum. **m** Immunoblot analysis of e-cadherin and vimentin. The results were expressed as relative expression to GADPH. **n** Immunostaining of e-cadherin and vimentin with SNAIL1 SiRNA. Scale bars = 200 μm. **o** NKX2–5-GFP hESCs was transfected with SiRNA and analyzed in an in vitro wound model. Scale bars = 200 μm. The percent wound closure (%) was counted under a light microscope. **p** Transwell assay in vitro. Migration cell was counted by DAPI in fluorescence microscope. Scale bars = 200μm. *n* = 3 each. Data are expressed as means ± SD. *P < 0.05, **P < 0.01 vs. control

Transcriptional profiling revealed 8 out of 11 known major EMT-associated genes were significantly upregulated or downregulated by treatment with UA at early stages of development, including SNAIL family members SNAI1 and SNAI2, and TWIST family members TWIST1 and TWIST2 (Fig. 6a).

The downregulation of SNAI1, SNAI2, TWIST1, and TWIST2 is consistent with evidence that TP53 downregulates TWIST and SNAI expression. On the expression of EMT marker genes, VIM was downregulated, whereas CDH1 and CDH2 were upregulated. The results were opposite to the characteristics of EMT. We speculated that UA could promote mesoderm development by promoting EMT in a short and precise window for early development. The expression of MIXL1 and GSC increased with UA treatment for 24 h compared with controls, whereas the EMT inhibitor GN-25 could abort the effect of mesoderm differentiation, resulting in decreased expression of MIXL1, GSC (Fig. 6b). As for the development of mesoderm, UA-treated hESCs showed a markedly increased percentage of BrdU^+^/MIXL1^+^ and BrdU^+^/GSC^+^ cells, whereas these promoting effects were fully abrogated by GN-25 treatment (Fig. 6c).

Previous studies have indicated UA may activate the WNT signaling pathway to increase beta-catenin. This in turn could increase SNAIL and TWIST to promote epithelial–mesenchymal transition (EMT) [9–11]. Therefore, we examined beta-catenin expression, and found that beta-catenin was increased by UA treatment after 24 h (Fig. 6d, e). We further assessed the temporal expression of vimentin (VIM), E-cadherin (CDH1), SNAIL, SLUG, TWIST1, and TWIST2 at the early stages of differentiation (Fig. 6f, S3A). Results revealed that VIM, SNAI1, TWIST1, and TWIST2 were upregulated and CDH1 and SNAI2 were downegulated at 24 h after UA treatment (Fig. 6f, S3A). This is consistent with previous reports of EMT-mediated mesoderm progenitor differentiation, (the earliest stage of differentiation in vitro) [12]. In these EMT genes, the expression of SNAIL was higher than others, which might have a great effect on EMT. Therefore we choose GN-25, inhibitor of interaction between SNAIL and TP53, to inhibit the function of SNAI. GN-25 downregulated VIM and upregulated CDH1. As expected SNAI1 did not change because GN-25 disrupts the interaction of SNAIL and TP53, rather than the SNAI1 expression (Fig. 6f, S3A). We then confirmed the protein level change of (VIM and CDH1) by western blot and immunostaining (Fig. 6g–k). Importantly, GN-25 also suppressed CDH1 compared with controls, suggesting impaired EMT progression. As enhanced migration is also an important phenotype of EMT, we tested the effect of GN-25 on cellular migration. As expected, the UA group exhibited a higher migratory activity and was able to heal the wound within 24 h compared with control, whereas GN-25 aborted the effect of UA with no significant cellular migration (Fig. 6i). Furthermore, the increased migration by UA and abrogation by GN-25 was further confirmed by transwell assays (Fig. 6j). As we had already suppressed the function of upstream molecule SNAI in EMT with GN-25, we next applied SiRNA to silence SNAI and confirmed successfully knockdown by qPCR (Fig. 6h). We then assessed EMT genes VIM and CDH1 by qPCR (Fig. 6l), and vimentin and e-cadherin by western blot and immunostaining (Fig. 6m, n). The results show that SNAI knockdown increases CDH1 and decreases VIM at transcriptome and protein levels. We also further performed cellular migration assays after SNAI knockdown (Fig. 6o, p).

UA promoted ubiquitin-mediated degradation of protein in UA-induced cardiomyocyte differentiation. SNAI can degrade by ubiquitination in EMT [13]. However, we found that UA-promoted EMT occurs ~ 24 h. AS the ubiquitination process takes effect from 24–48 h, we conclude MG-132 does not regulate EMT and that other regulators have greater importance during UA-induced cardiac differentiation before 24 h (Figure S3C).

### UA supports cardiogenesis by promoting mesoderm specification through ubiquitination within 24–48 h

**Fig. 7:**
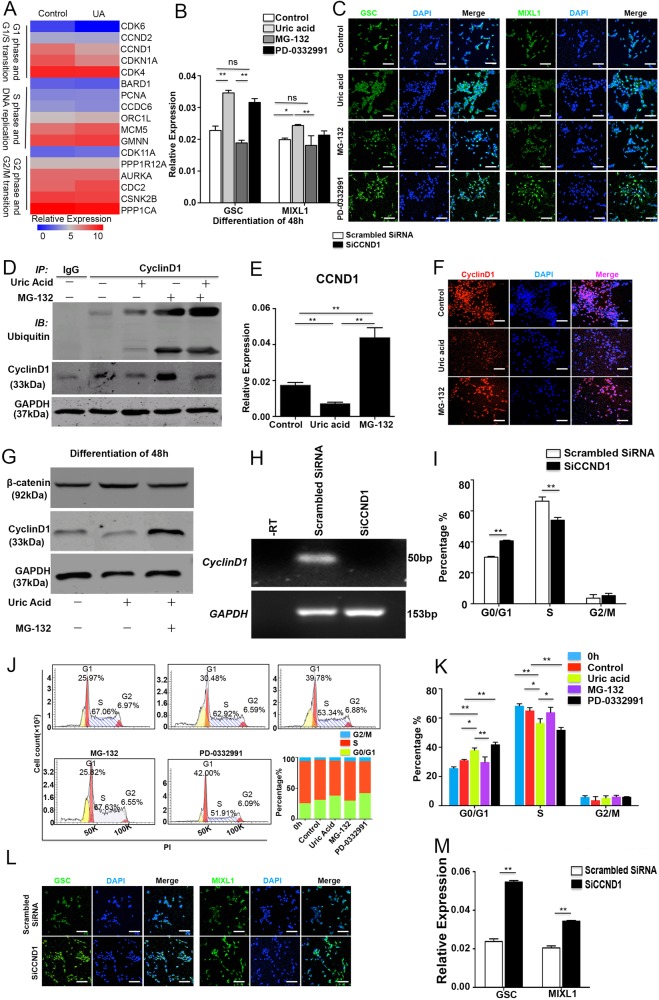
UA augments the cardiomyocyte population derived from hESCs by specifically promoting the proliferation of mesoderm cells by ubiquitination. **a** Heatmap shows hierarchical clustering of cell cycle genes. **b** The expression of mesoderm genes, GSC and MIXL1, were examined with quantitative PCR with or without UA, MG-132, and PD-0332991. The results were expressed as relative expression to GADPH and plotted as percentages of the maximum. **c** Immunostaining of GSC or MIXL1 in 48 h. Scale bars = 200 μm. **d** Immunoprecipitation analysis of cyclinD1 and beta-catenin. The results were expressed as relative expression to GADPH. **e** The expression of CCND1 was examined with quantitative PCR with or without UA and MG-132. The results were expressed as relative expression to GADPH and plotted as percentages of the maximum in UA treatment for 48 h. **f** Immunostaining of cyclinD1 with or without UA and MG-132 for 48 h. Scale bars = 200 μm. **g** Immunoblot analysis of cyclinD1 and beta- catenin. The results were expressed as relative expression to GADPH. **h** Gel electrophoresis with CCND1 SiRNA. The results were expressed as relative expression to GADPH. **i** Percentage of G0/G1 phase decreased in CCND1 SiRNA. **j** Cells were cultured for 0 h and 48 h with or without UA and MG-132 treatment. Representative flow cytometry histograms were performed with PI. Cell cycle analysis was performed using Modfit. **k** UA increases cell population in G0/G1 phase on 48 h. **l** Immunostaining of e-cadherin and vimentin with CCND1 SiRNA. Scale bars = 200 μm. **m** The expression of mesoderm genes, GSC and MIXL1, were examined with quantitative PCR with CCND1 SiRNA. The results were expressed as relative expression to GADPH and plotted as percentages of the maximum. *n* = 3 each. Data are expressed as means ± SD. *P < 0.05, **P < 0.01 vs. control

**Fig. 8:**
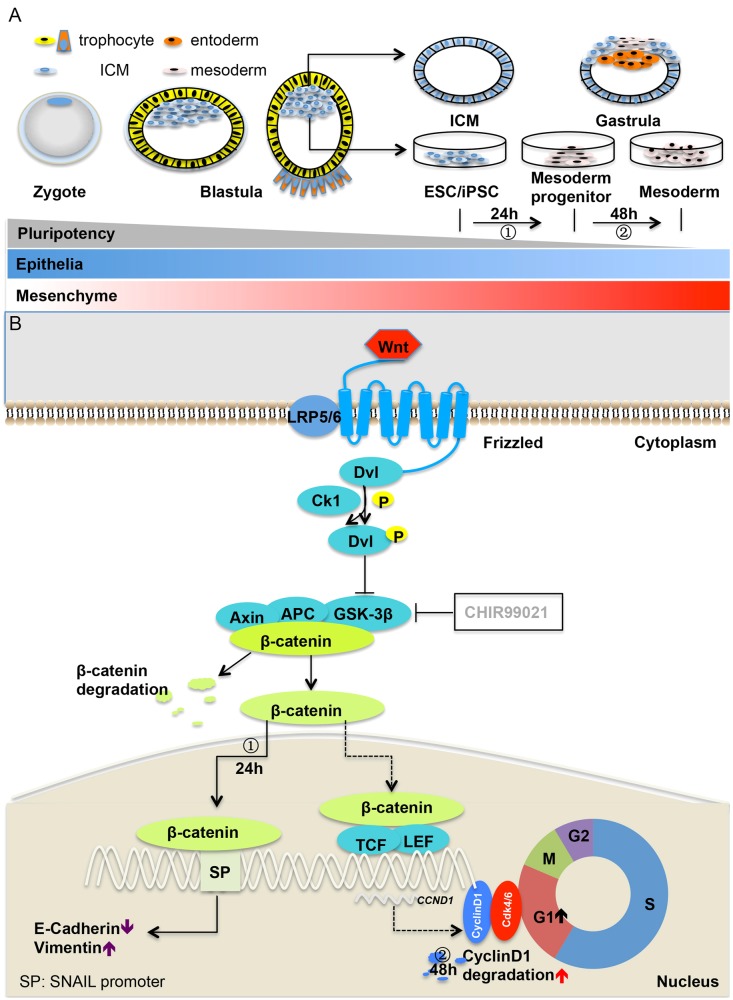
**a** Zygote is the beginning of embryonic differentiation. ESC in vitro comes from inner cell mass (ICM), which would development into gastrula with pluripotency gradually declined. Differentiation of ESC/iPSCs generates mesodermal characterized cells, including mesoderm progenitor and mesoderm. Epithelial–mesenchymal transition takes place in the development of gastrula. **b** ① Within 24 h UA could stimulate WNT pathway to increase beta- catenin and then promote EMT, downregulated E-cadherin and upregulated vimentin. ② UA could increase degradation of cyclinD1 in 48 h, which could decrease the expression of cyclinD1 and then lengthen G0/G1 phage. G0/G1 phage lengthened promoted differentiation

Through pathway analysis, we identified that UBC gene and ubiquitination processes may be important modulators of the early mesoderm development. Previous studies reported that the ubiquitination process promotes embryo development by regulated cyclinD1 in the cell cycle [14, 15]. We performed co-immunoprecipitation to clarify that UA could increase degradation of protein by ubiquitination. Results indicate that UA can increase degradation of cyclinD1 in 48 h, and decrease expression of cyclinD1 (Fig. 7d). As MG-132 is a specific dipeptide proteasome inhibitor that inhibits 26 s proteasome activity in the ubiquitin proteasome pathway [16], we then examined the effects of MG-132 on mesoderm. The most critical stage for UA effect is day 0–2 (Fig. 4c), the crucial phase for mesoderm specification. The expression of MIXL1 and GSC increased with UA treatment for 48 h compared with controls, whereas MG-132 aborted mesoderm specification indicated by decreased MIXL1 and GSC (Fig. 7b, c). As lengthened G1 phase indicates differentiation status [17, 18], we next assessed the impact of MG-132 on cell cycle. To further explore the effect of UA and MG-132 on cell cycle, we assessed the cell cycle profiles by flow cytometry. UA treatment significantly increased the percentage of G0/G1 stage cells and decreased the percentage of G2/M stage cells, and this effect was reversed with MG-132 treatment (Fig. 7j, k). These results show that UA can lengthen G0/G1 stage. Downregulation of cyclinD1 may induce cardiomyocytes differentiation with UA. Therefore, we examined the expression of CCND1 at different stages (Figure S3B). We found CCND1 RNA was significantly decreased with UA treatment for 24–48 h and increased with MG-132 treatment (Fig. 7e). CyclinD1 protein was consistently decreased on immunostaining and western blots (Fig. 7f, g). In order to show the effect of cyclinD1, we used PD-0332991 (inhibitor of CKD4) to examine mesoderm genes and protein by qPCR and immunofluorescence, respectively. We found GSC and MIXL1 expression was increased (Fig. 7b, c) and that PD-0332991 can prolong G0/G1 phase (Fig. 7j, k). In order to identify the effects of cyclinD1 in UA-induced cardiomyocyte differentiation, we used siRNA to knockdown CCND1 (Fig. 7h). GSC and MIXL1 expression increased as shown in qPCR and immunofluorescence data (Fig. 7l, m). Moreover, G0/G1 phage was shortened when CCND1 was silenced (Fig. 7i). Our results show no difference in CTNNB1 (Fig. 6d) and beta-catenin (Fig. 7g) expression at 48 h between UA and controls and that MG-132 had little effect on beta-catenin. We feel cyclinD1 is increased because of ubiquitination degradation rather than CCND1 transcriptional regulation. (Fig. 8)

## Discussion

Our study shows that UA can support cardiac differentiation of hESCs and hiPSCs by facilitating mesoderm differentiation. This reveals a novel cardiogenesic mechanism. Our major findings are (i) UA shows consistent cardiogenesic differentiation capacity in different hESCs and hiPSCs stem cells lines; (ii) UA can promote EMT to increase mesoderm differentiation in 0–24 h; (iii) Effects of UA are restricted to mesoderm phase of differentiation and this is owing to the lengthened G1 phase of cell cycle through ubiquitination between 24 and 48 h of differentiation (Fig. 8).

At present, cardiac differentiation induced by human stem cells in vitro is being explored as a model for cardiac development in vivo [7]. Differentiation at 0–2 day indicates stem cell differentiation into lateral mesoderm, then into the heart progenitor cells by 2–4 days and finally into cardiomyocyte by 4–6 days [19, 20]. Here, we confirm that UA promotes cardiac differentiation of hPSCs and we hypothesize that UA abnormalities in the early stages of embryonic development may be a risk factor for abnormal heart development. However, more clinical studies are needed to confirm the significance of the UA abnormalities as a predictor of CHD.

Previous studies have demonstrated that UA serum levels in humans have antioxidant properties [21–23]. Therefore, we tested whether the pro-cardiogenic effect of UA is due to its ability in decreasing ROS levels [24–29]. Our results show that typical antioxidants (including GMEE and NAC) fail to mimic the cardiomyocyte-promoting role of UA in stem cells. This is consistent with previous observations showing the inability of alternative antioxidative agents to mimic the effect of AA on the cardiac differentiation of hESCs [8, 30]. These findings suggest that the pro-cardiogenesic effect of UA is independent of its antioxidative properties, or at least, that its antioxidative effect is insufficient to facilitate cardiac differentiation of hPSCs.

Transition between epithelial features and mesenchymal counterparts have been well described during differentiation [31]. During morphological transition, cells obtain an ability to migrate enabling organogenesis after which de-mobility enables the cells to colonize appropriate sites for end organ development. The phenomenon that epithelial cells acquire mesenchymal traits, termed as epithelial–mesenchymal transition (EMT), has been observed in physiological and pathological processes, including embryogenesis, inflammation, fibrosis, wound healing, and cancer progression [32–34]. Loss of E-cadherin expression is considered a key event in EMT where the cell-cell contacting modulators, cell polarity-related cytoskeleton, and extracellular matrix are involved [35]. The Snail family of transcriptional repressors plays a critical role in regulating EMT [36, 37]. Snail1 and Snail2 bind the promoter of CDH1, encoding E-cadherin, to repress its transcription [37, 38]. Inhibiting SNAI1/P53 inhibited the proliferation of mesodermal cells, and also significantly decreased the migration ability of the cells.

It is believed that ES cells start to differentiate during G1 phase. A link between the G1 phase and commitment of ES cells into differentiation has been described [17, 18, 39]. In our study, expression of cyclinD1 decreased after UA treatment, leading to prolonged G1 phase in the cell cycle. The expression of endoderm increased, suggesting stem cell differentiation accompanied with mesodermal cell development. Ubiquitination plays an important role in the transition of each cell cycle. Many studies have shown that the ubiquitination pathway is involved in regulating the transition from G1 phase to S phase in the cell cycle [40]. CyclinD1 binds and activates cyclin-dependent kinase CDK4 to phosphorylate inhibitory protein Rb protein in Gl phase [41, 42]. The results of our RNA-seq suggest that genes of G1 phase downregulate and S and G2 upregulated. Cell cycle regulation may play an important role in UA-induced mesoderm differentiation. The novelty of our data is to link the cell cycle to the control of mesoderm fate and to show that the ubiquitination pathway is activated by UA regulating cyclinD1. This plays an important role in stimulating differentiation of mesoderm derived from PSCs. The expression of cyclinD1 is increased when ubiquitination is inhibited by MG-132, which promotes G1 phase into S phase. The prolonged S phase is accompanied with mesodermal gene and protein reduction, suggesting that UA lengthens G1 phase by increasing the degradation of protein by ubiquitination.

In summary, our findings demonstrate that UA robustly enhances the cardiac differentiation of both hESCs and hiPSCs. In addition, we show that UA promotes mesoderm formation via EMT by regulation of transcription factor SNAI1 at 0–24 h and the cell cycle control by increasing ubiquitination at 24–48 h. This is a novel and significant potential mechanism underpinning in vitro expansion of mesoderm in PSCs. Our study indicates that UA may play a critical role in heart development and may be a novel marker for CHD detection to facilitate early intervention.

## Methods and materials

### Reagents

Urate (uric acid sodium salt) (Sigma-Aldrich, St. Louis, MO, USA) was dissolved in 10 mmol/L NaOH. It was administered to cells at the final concentration of 2.5 mg/dl, 5 mg/dl, 7.5 mg/dl, 10 mg/dl, 20 mg/dl, and 30 mg/dl.

### PSCs culture

PSCs including hESCs-NKX2–5-GFP, hESCs-H9, and skin fibroblast-derived iPSCs (SFiPSCs, generated in our laboratory) were cultured without feeder cells in PSCeasy hESCs/iPSCs medium (modified essential 8 medium (Cellapy, Beijing, China)) (PSCeasy, DMEM/F12, L-ascorbic acid-2-phosphate magnesium (120 mg/L), sodium selenium (14 μg/L), β-FGF (100 ng/mL), insulin (19.4 mg/L), transferrin (10 mg/L), and TGFβ1 (2 μg/L)) on six-well plates (Corning Incorporated, Corning, NY, US) coated with a 1:200 dilution of growth factor-reduced matrigel (9 μg/cm^2^, Corning). Cells were passaged every 3–4 days with 0.5 mM ethylenediaminetetraacetic acid (EDTA) (Cellapy). Medium was changed every day. hESC lines were converted to E8/EDTA-based culture for at least five passages before the beginning of differentiation. All cultures were maintained with 2 mL medium per 9.6 cm^2^ of surface area or equivalent. All cells were maintained at 37 °C, 21% O_2_ and 5% CO_2_ in a humidified incubator (Thermo Fisher Scientific, Waltham, MA, USA).

### Cardiac differentiation of PSCs with uric acid

When PSCs were grown for 2 days, at which time they reached 70~80% confluence, namely at day 0 of induction of differentiation, adherent cells were washed with PBS (Hyclone, South Logan, UT, USA), and then enzymatically dissociated using 0.5 mM EDTA. Cells were seeded at a density of 8000–13,000 cells/cm^2^ in 1:200 growth factor-reduced matrigel (9 μg/cm^2^)-coated 12-well plates. Medium was changed to CDM3-UA, consisting of RPMI 1640 (Life Technologies Corporation, Gaithersburg, MD, USA), 500 μg/mL Oryza sativa-derived recombinant human albumin (Sigma-Aldrich, St. Louis, MO, US), and 7.5 mg/dl UA (Sigma-Aldrich). Medium was changed after every 48 h. For d0–d2, medium was supplemented with 6 μM CHIR99021 (Selleck Chemicals, Houston, TX, USA). On d2, medium was changed to CDM3-UA supplemented with 2 μM Wnt-C59 (Selleck Chemicals). Medium was changed on d4 and every day for CDM3-UA. Cardiomyocyte was derived from NKX2–5-GFP hESCs using the cardiac differentiation process above. When we further identified the effect of AA in cardiac differentiation, UA was replaced with 213 μg/mL L-ascorbic acid-2-phosphate magnesium (Selleck Chemicals) in the medium CDM3-AA during experimental procedures, and other differentiation procedure was retained.

### Flow cytometry and cell analysis

Differentiated NKX2–5-GFP hESCs at different time points were dissociated with Accutase (Innovative Cell Technologies, San Diego, CA, USA) for 30 min, and passed through a 30 μm pre-separation filter (Miltenyi Biotec Inc., Auburn, CA, USA) to produce a single cell suspension. The cells were analyzed on EPICS XL (Beckman Coulter, Miami, FL, USA) with Expo32 ADC XL 4 Color software. Data analysis was performed using FlowJo Software (Version X; TreeStar, Ashland, OR, USA). Cell sorting was performed using Fluorescence Activated Cell Sorting (FACS) on EPICS XL (Beckman Coulter).

NKX2–5-GFP ES cells were treated with MG-132 at specific concentrations (25 μM) and with UA (7.5 mg/dl) for 48 h. The collected cells were then fixed using cold 70% ethanol at −20 °C over 24 h, washed with PBS and incubated with 50 μg/mL propidium iodide (Becton, Dickinson and Company, Franklin lake, NJ, USA) at room temperature for 15 min. After being supplemented with propidium iodide, cycle cell analysis was performed using EPICS XL (Beckman Coulter). Percentage of cells at each cell cycle phases was determined with Modfit LT Software.

### Histology and immunohistochemical staining

Cardiomyocytes were plated onto growth factor-reduced Matrigel (9 μg/cm^2^, Corning)-coated 12-well plates (Corning) and were allowed to grow for 1–2 days. Immunostaining assays were performed according to the protocol. Cells were fixed with 4% PFA (Solarbio Science & Technology Co., Ltd, Beijing, China) for 30 min at room temperature, permeabilized with 0.1% Triton-X (Sigma-Aldrich) for 10 min at room temperature, blocked in 3% bovine serum albumin (Solarbio) for 30 min at room temperature and then incubated with primary antibodies against TNNI2 (1:100; Santa cruz Biotechnology, Inc., Santa Cruz, CA, USA), MYL2 (1:100; Abcam, Cambridge, MA, USA), MYL7 (1:100; Abcam), CX43 (1:100; Santa cruz Biotechnology), vimentin (1:200; Santa Cruz Biotechnology), and e-cadhrin (1:200; Santa cruz Biotechnology) overnight at 4 °C and detected by Alexa Fluor 488 goat anti-mouse IgG H & L and Alexa Fluor 647 goat anti-rabbit IgG H & L conjugated secondary antibodies. Nuclei were stained with Fluoroshield Mounting Medium with DAPI (Abcam) and staining with normal goat serum was used to be a negative control. Images were captured by Leica DMI 4000B fluorescence microscope or Leica TCS SP5 MP confocal laser scanning microscope (Leica, Wetzlar, Germany).

### RT-PCR and quantitative qRT-PCR

Cells were harvested with TRIzol™ Reagent (Invitrogen Inc. Carlsbad, CA, USA) and stored at −80 °C until use as per manufacturer’s instructions. cDNA was prepared using the GoScript™ Reverse Transcription System (Promega, Madison, WI, USA). QPCR was performed and analyzed by kinetic real-time PCR using the BIO-RAD (CFC Connect™, Hercules, CA, USA) with SYBR® Premix Ex Taq™ Tli RNaseH Plus (TaKaRa Biotechnology, Tokyo, Japan) for relative quantification of the indicated genes in triplicates. Gel electrophoresis was performed on the amplified products. The transcript of glyceraldehyde phosphate dehydrogenase (GADPH) was used for internal normalization. The qRT-PCR primers are listed in Supplementary information Table S1.

### Cell proliferation assays

Cell proliferation was assayed by the Cell Counting Kit-8 (CCK-8) assay (Dojindo, Kumamoto, Japan) according to the manufacturer’s protocol. The NKX2–5-GFP hESCs were plated in 96-well plates (2000 cells/well). Following the manufacturer’s protocol, cell proliferation was detected every 24 h. In brief, 10 µl of CCK-8 solution was added to each well and incubation was carried out for 1.5 h at 37 °C. Then, each solution was measured spectrophotometrically at 450 nm (Bio-Tek Synergn 4, Winooski, VT, USA).

Proliferation status of the cells was determined by measuring the incorporation of BrdU. Cells were incubated with 10 μmol/L BrdU (Abcam) for 24 h or 48 h and BrdU labeling was detected by confocal laser scanning microscope using an Alexa Fluor 647 conjugated anti-BrdU antibody (1:50; Abcam), following the immunostaining protocols. Staining of samples without BrdU was used as negative control. Double staining of BrdU with GSC and MIXL1 was performed by GSC (1:100; Abcam) and MIXL1 (1:100; Proteintech, Rocky Hill, NJ, USA) antibody.

### Immunoblot and co-immunoprecipitation analysis

Immunoblot analyses were performed according to the protocol. Protein samples were size fractionated by sodium dodecyl sulphate-polyacrylamide gel electrophoresis and the separated proteins were electrophoretically transferred to hybridization nitrocellulose filter (Merck Millipore ltd., Billerica, MA, USA). Then the membrane was incubated with primary antibodies against vimentin (1:1000; Santa Cruz Biotechnology), e-cadherin (1:1000; Santa Cruz Biotechnology), cyclinD1 (1:1000; Abcam), beta-catenin (1:1000; Santa Cruz Biotechnology) and GADPH (1:1000; Santa Cruz Biotechnology). Horseradish peroxidase-linked anti-rabbit (1:4000; Santa Cruz Biotechnology) or anti-mouse (1:4000; Santa Cruz Biotechnology) was used as secondary antibodies. To determine cyclinD1 and ubiquitin interaction, immunoprecipitation was performed with anti-cyclinD1 antibody (1:30; Abcam). In brief, cell extracts were incubated with cyclinD1 at 4 °C overnight, respectively, and then mixed with protein A/G agarose beads (Sangon) at 4 °C overnight. Beads were washed, and proteins pulled down were analyzed using anti-ubiquitin (1:1,000; Santa Cruz Biotechnology) to detect cyclinD1 by western blotting described previously.

### Cell invasive assays

NKX2–5-GFP hESCs were removed by 0.5 mM EDTA, counted and plated at 46,105 cells/mL in 12-well dishes. Cells were incubated 2 days yielding confluent monolayers for wounding, and then cells were treated as described above. Wounds were made using a pipette tip and photographs taken immediately (time zero) and 24 h after wounding for NKX2–5-GFP hESCs with GN-25 and UA or not, respectively. The distance migrated by the cell monolayer to close the wound area during this time period was measured by image J. Results were expressed as a migration or targeted relative to the scratch area migrated by UA-treated cells. Transwell migration assays on Matrix gels were carried out in duplicate samples. The cells in the lower compartment and those in the lower surface of the filter were collected after 8 h of incubation and counted. In a parallel experiment, the nuclei of the cells present in the lower surface of the filters were stained with 4,6-diamidinophenylindole (DAPI) after fixing in methanol and careful removal of the cells present in the upper surface of the filters. Experiments were carried out in triplicate and repeated at least three times.

### siRNA and transfection

To study the effect of SNAIL and CCND1 silencing, we utilized siRNA technology. SNAI1 or CCND1 siRNA and control siRNA were obtained from Sigma. Cells were transfected with either control siRNA or SNAI1, CCND1 siRNA for 24 h using Lipofectamine 2000 (Invitrogen) according to the manufacturer’s protocol. After the siRNA treatment period, knockdown efficiency was evaluated by PCR analysis.

### Total RNA extraction

RNA was extracted using Trizol at 0 °C, and the quantity were determined by 2% agarose gel electrophoresis. The mRNA was enriched by using the oligo (dT) magnetic beads. The double strand cDNA was synthesized and sequencing adaptors was ligated to the fragments. The fragments are enriched by PCR amplification. Agilent 2100 Bioanaylzer and ABI StepOnePlus Real-Time PCR System are used to qualify and quantify of the sample library. (Table 1)

**Table S1:** Basic clinical characteristics of CHD

	Non-CHD (535)	CHD (306)	*P* value
Year (mean ± SD)	26.9 ± 3.72	30.33 ± 4.69	0.00
Diabetes (*N*)	32	28	0.09
Gravidity NO (mean ± SD)	1.47 ± 0.32	1.44 ± 0.35	0.73
Hyperuricemia (*N*)	37	43	0.04

Mean ± SD, mean ± standard deviation, *CHD* congenital heart disease, *NO* number

### Processing of transcriptome sequencing and bioinformatics analysis

mRNA sequencing was performed on the Illumina HiSeq™ 2000. Base calling was adopted to convert original sequencing images to sequential data as the raw reads. The raw reads were subjected to adapter trimming and low-quality filtering using Trimmomatic program. The high-quality clean reads were aligned to the human genome using Bowtie2. Human genome (hg19) sequence and gene annotation were obtained from the UCSC Genome Website (http://genome.ucsc.edu/). NOISeq was used to profile differentially expressed genes with default parameters. Gene ontology (GO) functional enrichment analysis was completed using the web-based GO analysis tool, WEGO (http://wego.genomics.org.cn/cgi-bin/wego/index.pl). WEGO was used to map all differentially expressed genes (DEGs) and to search for significantly enriched GO terms in DEGs compared with the genomic background. Pathways were constructed using the KEGG database, following the custom scripts. KEGG pathway enrichment analysis of DEGs compared with transcriptome background was performed by hypergenometric distribution testing using the Phyper function of the R software package (http://www.r-project.org/). Bonferroni correction was used to adjust *P* value for each pathway. Different proteins often form complex proteins through complicated interactions to perform their biological functions. Protein–protein interaction network analysis was performed by cytoscape version 3.4.0.

### Statistical analysis

All experiments were performed at least three times, and data were expressed as mean ± standard deviation and analyzed by Student’s *t* test or one-way analysis of variance with post hoc analysis. A value of *P* < 0.05 was considered statistically significant.

## Electronic supplementary material

Supplementary Legends

Fig-S1

Fig-S2

Fig-S3

Table S2

Table S3

Movie 1
