# Supplementary material for: Uric acid: a potent molecular contributor to pluripotent stem cell cardiac differentiation via mesoderm specification
Source: Cell Death Differ. 2018 Jul 23;26(5):826–42. doi: 10.1038/s41418-018-0157-9 (PMC6461775; doi:10.1038/s41418-018-0157-9)
Supplement: Supplementary file 1 — Supplementary Legends [file 41418_2018_157_MOESM1_ESM.docx]

**Supplementary Legends**

**Figure S1**: (A, B) Schematic diagram of AA-induced cardiac differentiation protocols. Efficiency of cardiac differentiation measured by flow cytometry for EGFP+ on day 15 cells in different differentiation protocols. (C) Efficiency of cardiac differentiation measured by flow cytometry for EGFP+ with AA, UA and AA+UA. (D) The expression of mesoderm genes, GSC and MIXL1, were examined with Quantitative PCR with or without AA at 0-2d. The results were expressed as relative expression to GADPH and plotted as percentages of the maximum. (E) The expression of genes of VIM, CDH1 and CCND1 were examined with Quantitative PCR with or without AA at 0-2d. The results were expressed as relative expression to GADPH and plotted as percentages of the maximum. n= 3 each. Data are expressed as means ± SD. *P < 0.05, **P < 0.01 *vs.* control.

**Figure S2:** Heatmap shows hierarchical clustering of pluripotent, endoderm, mesoderm and ectoderm genes. Values are row-scaled to show relative expression. Blue and red are low and high levels respectively. Representative down- (blue box) and upregulated (red box) genes are listed.

**Figure S3:** (A) The expression of EMT genes, VIM, CDH1, SNAI1, SNAI2, TWIST1 and TWIST2, were examined with Quantitative PCR with or without UA for every 12h. The results were expressed as relative expression to GADPH and plotted as percentages of the maximum. (B) The expression of CCND1 was examined with Quantitative PCR with or without UA for every 12h. The results were expressed as relative expression to GADPH and plotted as percentages of the maximum. (C) Immunoblot analysis of e-cadherin and vimentin with or without UA and MG-132 in 24h. The results were expressed as relative expression to GADPH. (D) Cell viability was measured by CCK8 in different concentration of MG-132. (E) Schematic diagram of AA or UA induced cardiac differentiation protocols in 0-4d. (F) NKX2-5-GFP hESCs were treated with 7.5mg/dl UA at 0-2d or 2-4d respectively. Control group was treated without either AA or UA. Scale bars =200μm. (G) The expression of CPC specification genes, NKX2-5 and TBX-5 were examined with Quantitative PCR. The results were expressed as relative expression to GADPH and plotted as percentages of the maximum. n= 3 each. Data are expressed as means ± SD. *P < 0.05, **P < 0.01 *vs.* control.
