# Supplementary material for: Uric acid: a potent molecular contributor to pluripotent stem cell cardiac differentiation via mesoderm specification
Source: Cell Death Differ. 2018 Jul 23;26(5):826–42. doi: 10.1038/s41418-018-0157-9 (PMC6461775; doi:10.1038/s41418-018-0157-9)
Supplement: Supplementary file 5 — Table S2 [file 41418_2018_157_MOESM5_ESM.docx]

**Supplementary information, Table S1** Primers used for RT-PCRs

| Gene | Primer Sequence (5' to 3') | AT (°C) |
| --- | --- | --- |
| *NKX2-5* | F-CTGTCTTCTCCAGCTCCACC | 59.5 |
|  | R-TTCTATCCACGTGCCTACAGC | 57.6 |
| *GATA4* | \| F-TACATGTCTCTCCCCTGGCA \| \| --- \| | 57.4 |
|  | R-GAACGAAGGGTCTGCAGTGA | 57.4 |
| *MYH7* | F-CGCACCTTCTTCTCTTGCTC | 57.4 |
|  | R-GAGGACAAGGTCAACACCCT | 57.4 |
| *MYH6* | F-CTTCTCCACCTTAGCCCTGG | 59.5 |
|  | R-GCTGGCCCTTCAACTACAGA | 57.4 |
| *TNNT2* | F-GCGGGTCTTGGAGACTTTCT | 57.4 |
|  | R-TTCGACCTGCAGGAGAAGTT | 55.4 |
| *VIM* | F-GCTTCAGAGAGAGGAAGCCG | 59.5 |
|  | R-AAGGTCAAGACGTGCCAGAG | 57.4 |
| *CCND1* | F-TTCAGCCTGTTTGGCGTTTC | 55.4 |
|  | R-ACCCATGCCTGTCCAATCAG | 57.4 |
| *SNAI1* | F-GGCCTAGCGAGTGGTTCTTC | 59.5 |
|  | R-TTCCTGACGAGGAAAGAGCG | 57.4 |
| *CDH1* | F-GTCGAGGGAAAAATAGGCTG | 55.4 |
|  | R-GCCGAGAGCTACACGTTCAC | 59.5 |
| *SNAIL2* | F-ATCACTGTGTGGACTACCGC  R-GAGAGGCCATTGGGTAGCTG | 59.75  60.18 |
| *TWIST1* | F-TTCAAAGAAACAGGGCGTGG  R-CCGTCTGGGAATCACTGTCC | 58.97  60.11 |
| *TWIST2* | F-TGAAACCTGAACAACCTCAGGA  R-CTGTCCCTTCTCTCGACGC | 59.49  59.86 |
| *CTNNB1* | F-ATAAGAGCTCCTTGTGCGGC  R-CCTCAGACCTTCCTCCGTCT | 60.46  60.32 |
| *SOX2* | F-GGGAAATGGGAGGGGTGCAAAAGAGG | 64.3 |
|  | R-TTGCGTGAGTGTGGATGGGATTGGTG | 62.8 |
| *OCT3/4* | F-GACAGGGGGAGGGGAGGAGCTAGG | 68.1 |
|  | R-CTTCCCTCCAACCAGTTGCCCCAAAC | 64.3 |
| *ESRRB* | F-GAGATGCGCAGGTTAGGCTC | 59.5 |
|  | R-GCTTCCATTGCCAGTCCACA | 57.4 |
| *REX1* | F-CGCGGTAACAGGGACAAATGTA | 57.7 |
|  | R-AGCAAACACCTGCTGGACTG | 57.4 |
| *NES* | F-GACCCTGAAGGGCAATCACA | 57.4 |
|  | R-GGCCACATCATCTTCCACCA | 57.4 |
| *SOX1* | F-AATACTGGAGACGAACGCCG | 57.4 |
|  | R-AACCCAAGTCTGGTGTCAGC | 57.4 |
| *MIXL1* | F-TTTTCTCCCCTCTTCCAGGTAT | 57.4 |
|  | R-GGCCTAGCCAAAGGTTGGAA | 55.8 |
| *GSC* | F-TCCTCATCAGAGGAGTCGGA | 57.4 |
|  | R-ACGACGACGTCTTGTTCCAC | 57.4 |
| *SOX17* | F-GCCAGCTCCGCGGTATATTA | 57.4 |
|  | R-GGATCAGGGACCTGTCACAC | 59.5 |
| *GATA6* | F-CTAGACGTCAGCTTGGAGCG | 59.5 |
|  | R-CTGGAAAGGCTCTGGAGTCG | 59.5 |
| *FOXA2* | F-TGCACTCGGCTTCCAGTATG | 57.4 |
|  | R-CATGTTGCTCACGGAGGAGT | 57.4 |
| *AFP* | F-CATATGCCAACAGGAGGCCA | 57.4 |
|  | R-CTGAGCTTGGCACAGATCCT | 57.4 |
| *T*  *TBX5* | F-TGCTTCCCTGAGACCCAGTT | 57.4 |
|  | R-GATCACTTCTTTCCTTTGCATCAAG  F-ATGCAAGAGACCTCAGTCCC  R-CTCTTCACGAAGGGAGGTGG | 56.3  59.1  59.8 |
| *GADPH* | F-GTGGACCTGACCTGCCGTCT | 61.6 |
|  | R-GGAGGAGTGGGTGTCGCTGT | 61.6 |
