# Supplementary material for: Uric acid: a potent molecular contributor to pluripotent stem cell cardiac differentiation via mesoderm specification
Source: Cell Death Differ. 2018 Jul 23;26(5):826–42. doi: 10.1038/s41418-018-0157-9 (PMC6461775; doi:10.1038/s41418-018-0157-9)
Supplement: Supplementary file 6 — Table S3 [file 41418_2018_157_MOESM6_ESM.docx]

**Supplementary information, Table S2 *C*omparisons among differentiation stage**

|  | *P* value summary (***P* value < 0.01, **P* value< 0.05) |
| --- | --- |
| C01C02C03-UA(+) vs. C01-UA(+) | ns |
| C01C02C03-UA(+) vs. C02-UA(+) | ** |
| C01C02C03-UA(+) vs. C03-UA(+) | ** |
| C01C02C03-UA(+) vs. C01C02-UA(+) | ns |
| C01C02C03-UA(+) vs. C01C03-UA(+) | ** |
| C01C02C03-UA(+) vs. C02C03-UA(+) | ** |
| C01C02C03-UA(+) vs. C01C02C03-UA(-) | ** |
| C01-UA(+) vs. C02-UA(+) | ** |
| C01-UA(+) vs. C03-UA(+) | ** |
| C01-UA(+) vs. C01C02-UA(+) | ns |
| C01-UA(+) vs. C01C03-UA(+) | * |
| C01-UA(+) vs. C02C03-UA(+) | ** |
| C01-UA(+) vs. C01C02C03-UA(-) | ** |
| C02-UA(+) vs. C03-UA(+) | ns |
| C02-UA(+) vs. C01C02-UA(+) | * |
| C02-UA(+) vs. C01C03-UA(+) | ns |
| C02-UA(+) vs. C02C03-UA(+) | ns |
| C02-UA(+) vs. C01C02C03-UA(-) | ns |
| C03-UA(+) vs. C01C02-UA(+) | * |
| C03-UA(+) vs. C01C03-UA(+) | ns |
| C03-UA(+) vs. C02C03-UA(+) | ns |
| C03-UA(+) vs. C01C02C03-UA(-) | ns |
| C01C02-UA(+) vs. C01C03-UA(+) | ns |
| C01C02-UA(+) vs. C02C03-UA(+) | * |
| C01C02-UA(+) vs. C01C02C03-UA(-) | ** |
| C01C03-UA(+) vs. C02C03-UA(+) | ns |
| C01C03-UA(+) vs. C01C02C03-UA(-) | ns |
| C02C03-UA(+) vs. C01C02C03-UA(-) | ns |
